# Adverse Events Reporting in Digital Interventions Evaluations for Psychosis: A Systematic Literature Search and Individual Level Content Analysis of Adverse Event Reports

**DOI:** 10.1093/schbul/sbae031

**Published:** 2024-04-06

**Authors:** Stephanie Allan, Thomas Ward, Emily Eisner, Imogen H Bell, Matteo Cella, Imran B Chaudhry, John Torous, Tayyeba Kiran, Thomas Kabir, Aansha Priyam, Cara Richardson, Ulrich Reininghaus, Anita Schick, Matthias Schwannauer, Suzy Syrett, Xiaolong Zhang, Sandra Bucci

**Affiliations:** School of Health and Wellbeing, University of Glasgow, Glasgow, UK; School of Mental Health and Psychological Sciences, Department of Psychology Institute of Psychiatry, Psychology & Neuroscience, King’s College London, London, UK; South London & Maudsley NHS Foundation Trust, London, UK; Division of Psychology and Mental Health, School of Health Sciences, Faculty of Biology, Medicine and Health, Manchester Academic Health Sciences, The University of Manchester, Manchester, UK; Orygen, Parkville, VIC, Australia; Centre for Youth Mental Health, University of Melbourne, Parkville, VIC, Australia; School of Mental Health and Psychological Sciences, Department of Psychology Institute of Psychiatry, Psychology & Neuroscience, King’s College London, London, UK; South London & Maudsley NHS Foundation Trust, London, UK; Division of Psychology and Mental Health, School of Health Sciences, Faculty of Biology, Medicine and Health, Manchester Academic Health Sciences, The University of Manchester, Manchester, UK; Ziauddin University and Hospital Karachi, Karachi, Pakistan; Pakistan Institute of Living & Learning, Karachi, Pakistan; Department of Psychiatry, Beth Israel Deaconess Medical Center, Harvard Medical School, Boston, MA, USA; Centre for Youth Mental Health, University of Melbourne, Parkville, VIC, Australia; Department of Psychiatry, University of Oxford, Oxford, UK; Division of Psychology and Mental Health, School of Health Sciences, Faculty of Biology, Medicine and Health, Manchester Academic Health Sciences, The University of Manchester, Manchester, UK; Division of Psychology and Mental Health, School of Health Sciences, Faculty of Biology, Medicine and Health, Manchester Academic Health Sciences, The University of Manchester, Manchester, UK; School of Mental Health and Psychological Sciences, Department of Psychology Institute of Psychiatry, Psychology & Neuroscience, King’s College London, London, UK; Department of Public Mental Health, Central Institute of Mental Health, Medical Faculty Mannheim, Heidelberg University, Mannheim, Germany; Department of Psychiatry, University of Oxford, Oxford, UK; Department of Public Mental Health, Central Institute of Mental Health, Medical Faculty Mannheim, Heidelberg University, Mannheim, Germany; Department of Clinical and Health Psychology, School of Health in Social Science, University of Edinburgh, Edinburgh, UK; School of Health and Wellbeing, University of Glasgow, Glasgow, UK; Division of Psychology and Mental Health, School of Health Sciences, Faculty of Biology, Medicine and Health, Manchester Academic Health Sciences, The University of Manchester, Manchester, UK; Division of Psychology and Mental Health, School of Health Sciences, Faculty of Biology, Medicine and Health, Manchester Academic Health Sciences, The University of Manchester, Manchester, UK; Greater Manchester Mental Health NHS Foundation Trust, Manchester, UK

**Keywords:** psychosis, schizophrenia, adverse effects, digital health, safety

## Abstract

**Background:**

Digital health interventions (DHIs) have significant potential to upscale treatment access to people experiencing psychosis but raise questions around patient safety. Adverse event (AE) monitoring is used to identify, record, and manage safety issues in clinical trials, but little is known about the specific content and context contained within extant AE reports. This study aimed to assess current AE reporting in DHIs.

**Study Design:**

A systematic literature search was conducted by the iCharts network (representing academic, clinical, and experts by experience) to identify trials of DHIs in psychosis. Authors were invited to share AE reports recorded in their trials. A content analysis was conducted on the shared reports.

**Study Results:**

We identified 593 AE reports from 18 DHI evaluations, yielding 19 codes. Only 29 AEs (4.9% of total) were preidentified by those who shared AEs as being related to the intervention or trial procedures. While overall results support the safety of DHIs, DHIs were linked to mood problems and psychosis exacerbation in a few cases. Additionally, 27% of studies did not report information on relatedness for all or at least some AEs; 9.6% of AE reports were coded as unclear because it could not be determined what had happened to participants.

**Conclusions:**

The results support the safety of DHIs, but AEs must be routinely monitored and evaluated according to best practice. Individual-level analyses of AEs have merit to understand safety in this emerging field. Recommendations for best practice reporting in future studies are provided.

## Introduction

People who experience psychosis continue to face significant barriers in accessing evidence-based care.^1^ Digital health interventions (DHIs) have been heralded to upscale access to treatment, and many are being trialed^2^ and implemented.^3^ DHIs can also offer access to timely support.^4^ Alongside the potential benefits of increased access, it is important to consider potential risks or harms. While some safety concerns are shared with other psychological and pharmacological interventions,^5^ such as potential symptom exacerbation, real-world engagement with DHIs in the absence of clinician monitoring requires unique consideration for safety monitoring. Before a health professional can recommend a stand-alone DHI (ie, delivered with no health professional input) or implement a blended intervention (ie, where digital tools are used in conjunction with face-to-face care), they need to know it is safe for patients to use. Equally, patients require accessible and trustworthy information about both the clinical benefits and potential harms to provide informed consent for treatment.

Best practice recommends that safety is evaluated at the clinical trial stage through routine monitoring of adverse events (AEs), including oversight from an independent monitoring committee and reported following established guidance designed for social and psychological interventions.^6,7^ The Good Clinical Practice guidelines of the International Council for Harmonization foreground the importance of monitoring AEs,^8^ defined as “any untoward medical occurrence in a patient or clinical investigation subject administered a pharmaceutical product and which does not necessarily have a causal relationship with this treatment. An AE is an unfavorable and unintended sign (including an abnormal laboratory finding), symptom, or disease temporally associated with the use of a medicinal (investigational) product, whether or not related to the medicinal (investigational) product” (p. 1).^9^ However, AEs can also be related to trial procedures such as a participant becoming distressed during an outcome assessment. AEs can be categorized with respect to “relatedness” (ie, whether there is evidence of any causal relationship between the AE and the intervention^10^) and “expectedness” (ie, whether the AE is consistent with the outcomes expected within a particular population as defined and identified by the research team when planning the study). AEs can be further categorized in terms of seriousness. The severity or intensity of the AE is typically categorized by researchers as either mild (an event tolerated by the patient that does not interfere with everyday activities), moderate (an event sufficiently discomforting to interfere with normal everyday activities), or severe (an event that prevents normal everyday functioning). Serious adverse events (SAEs) are defined in different legislation as a “death/life-threatening, hospitalization, disability, congenital disability, or a “medically important event” (p. 1)^9^ or leading to “chronic ill health”^11^ (p. 19).

While there is growing interest in the acceptability and feasibility of novel DHIs,^12^ concerns have been expressed regarding the conceptualization and reporting of AEs within trials of psychosocial interventions more generally,^13^ and in psychosis specifically,^14^ because standard definitions were developed for pharmacological trials.^15,16^ Such guidance is likely to be biased toward identifying “untoward medical occurrences” which privileges the monitoring of events that professionals view as crucial (typically hospitalization and medical intervention), while potentially neglecting adverse psychological and social effects that may be impactful to patients. Evaluation of relatedness of AEs may be driven by a patient’s unique perspective of the intervention,^17^ which may be especially pertinent for stand-alone DHIs. While monitoring AEs is standard practice in clinical trials of DHIs, a recent review highlighted significant issues with respect to transparency of reporting, in particular regarding relatedness.^18^ One strategy to address this is to develop safety monitoring tools, which are tailored to the unique aspects of DHIs and designed through a partnership between patients and professionals.

### Study Aims

While several AE frameworks exist,^19^ they are typically country-specific. Harmonization in the conceptualization, monitoring, and reporting of AEs is important to ensure the transparency, accessibility, and generalizability of findings and the utility of monitoring tools across diverse international settings. Digital health studies for psychosis are not monolithic but rather comprise a range of techniques and components leveraged to target varied problems, including, eg, cognitive problems,^20^ motivation deficits,^21^ and paranoia.^22^ Therefore, the informative question to answer is not “Are DHIs for psychosis safe?,” but rather “To what extent are different aspects of DHIs associated with potential harms, and how can these be mitigated?” To answer this, there is a need to build our understanding of relationships between aspects of the DHI (eg, functions, delivery mode, and therapeutic targets), and the frequency of AEs, including those that may be important to the patient but missed within standard trial reporting protocols. Content analysis of individual-level data (in the form of AE reports) presents a valuable opportunity for learning through considering contextual factors which are routinely collected for trial monitoring but are typically omitted in published manuscripts where data is limited to a narrow set of prespecified events (eg, hospital admissions, deaths). A standardized and widely used AE coding framework tailored to DHIs could facilitate precision and consistency in documenting and reporting AEs in DHI across trials. Presenting such a framework represents an important step in building evidence of what types of AEs commonly occur in the context of DHIs for psychosis. To address this, the aims of this study were: (1) collate individual-level records of AEs from published international digital health trials for psychosis; (2) develop a coding framework to enable content analysis of negative consequences contained within AE reports including associated contextual factors; (3) explore evidence of the relatedness of AEs to DHIs; and (4) map AE frequency data onto a typology delineating different DHI components.

## Methods

### Setting: The *iCharts Network*

The iCharts network (International Collaboration for Harmonizing Adverse Events Reporting in Technology for Serious Mental Health Problems) was formed through a Schizophrenia International Research Society’s 2021–2023 Research Harmonization Award to harmonize AE monitoring practices in digital psychosis research. iCharts is a group of international experts working on developing and evaluating DHIs for psychosis, including academics, clinicians, and experts by experience across 7 countries (United Kingdom, Belgium, Germany, Pakistan, Australia, United States, and China), including 2 low- or middle-income countries (Pakistan, China).

Through this group diversity, the network draws on both existing datasets and members’ international expertise, lived experience of psychosis perspectives, and considers cross-cultural issues and international differences. This paper presents the findings from the individual-level records of AEs reported across digital health studies in psychosis.

### Phase 1: Systematic Search of Relevant DHI Trials and Collation of Individual Level AE Data

Seven databases (MEDLINE, PsycINFO, PsycARTICLES, Embase, Health and Psychosocial Instruments, PubMed, and Web of Science) were systematically searched combining search terms relating to both psychosis/schizophrenia and digital health (see supplementary methods SMI for more detail including dates). Searches were restricted to English language reports involving peer review since January 2010 (because most DHI studies for psychosis have been published since that point). Inclusion criteria were studies testing the use of digital health tools that aim to monitor or improve the mental or physical health of people with a psychosis or schizophrenia spectrum diagnosis using a device such as a smartphone app, text messaging, online/website, virtual reality, or wearable device. Exclusion criteria were studies: (1) where digital tools were used as a component during in-person sessions with no independent use outside these sessions (except for Virtual Reality studies, which were included based on in-person use only); (2) that were designed purely for research purposes (with no likely eventual clinical application); (3) that only included video-conferencing or phone calls; (4) that served only as an appointment booking system for in-person therapy; (5) that were only for mental health staff to update electronic health records; (6) that were only used to screen for the presence of a mental health condition; and (7) harvested existing data from electronic health records or mainstream social media to make predictions or classifications of a mental health condition. Author EE combined the search results, removed duplicates, and screened titles and abstracts of the combined results against eligibility criteria. To ensure reliability, author CR independently screened the titles and abstracts of a randomly selected 10% sample of retrieved articles. Ratings were compared and disagreements were resolved by consensus. Two researchers then independently screened the full texts against PICO criteria (supplementary methods SM2). Ratings were then compared (*k* = −0.88), and any disagreements resolved by consensus.

Author SB contacted the corresponding author of all articles that met eligibility criteria citing the relevant article and requested a de-identified list of the type and nature of AEs in the study, with 2 reminder prompts sent at fortnightly intervals to authors, where needed (see supplementary materials SM3). The Schizophrenia International Research Society network also emailed their distribution list to request this information (no additional studies were identified via this route). Details of the standardized operating procedures used to monitor AEs were also requested; these were analyzed in a separate but related paper.^23^

In parallel, a secure online form allowed authors to share and upload relevant de-anonymized information. A data extraction form was developed to aggregate this raw data. Where an author responded to the email, a proforma was sent inviting authors to submit de-identified data from their trial AE reporting forms. Where studies included a qualitative component, we asked authors to extract relevant AE-related data from the interview transcript. Specifically, we requested information on which AEs were formally reported during the study, how many times each was reported, and whether these were related to study procedures or the intervention. Authors extracted and de-identified the relevant information locally to avoid breaching existing ethical agreements. Authors were required to confirm that: (1) only anonymous aggregated data will be shared; (2) data were gathered under an existing ethical agreement, according to the laws of the country where it was gathered; and (3) the necessary permissions were in place to share the relevant data. A data-sharing agreement was put in place where needed.

### Phase 2: Content Analysis

To analyze the raw AE reports to determine the type of information contained in AE events, a semi-inductive content analysis according to Hsieh^24^ was performed using a bespoke coding framework. This approach involves interpreting textual information by examining the explicit content (eg, the frequency of terms commonly associated with AEs such as psychiatric hospitalization) and the context within which these terms were presented (eg, context of a patient’s life). Owing to the exploratory nature of the analysis, we considered all AE reports regardless of when they were detected in the trial (eg, baseline, follow-up). While aiming to be inductive, we acknowledge that we may have been influenced by various AE reporting tools that we ourselves have used in previous work. This was discussed recursively throughout the coding process and for this reason the content analysis is framed as semi-inductive. After anonymization, we analyzed the individual-level AE data in the following stages:

1. raw individual-level AEs were collated from 18 DHI studies resulting in 593 AE reports. Within these data, it was not possible to distinguish between AEs associated with a DHI and those in a control condition;
2. a list of AEs was randomly assigned to 7 coders, including psychiatrists, psychologists, and patients, with career levels spanning from research assistants to professors, who were instructed to code the data inductively, which meant deciding if there was an adverse consequence in the AE report, and if so, to name this as an inductive thematic code;
3. authors SA and TW compared within and across the inductive codes generated in step 2, and developed a common set of codes. In line with the semi-inductive method, we added certain SAEs to the set of codes (eg, suicide, death) that rarely appeared in the original data set;
4. the coding framework was finalized through discussion with the wider research group and used to code the AEs. In line with the aims of understanding relevant contextual factors, a single reported AE could include multiple codes. For example, an individual sectioned in a psychiatric hospital (a single reported AE) precipitated by an increase in substance use and exacerbation of psychosis symptoms received 3 thematic codes (psychotic exacerbation, substance misuse, and psychiatric admission);
5. to determine the relative ratio of potential AE codes across a heterogeneous range of study designs, frequencies of specific codes were summed and divided by the overall number of AE codes identified for each intervention. For example, if an intervention yielded 10 codes during the content analysis stage, 3 of which were for hospital admissions, this would result in a score of 0.33, or 33%.

### Phase 3 Mapping AE Coded Onto a DHI Typology

**Fig. 1.**
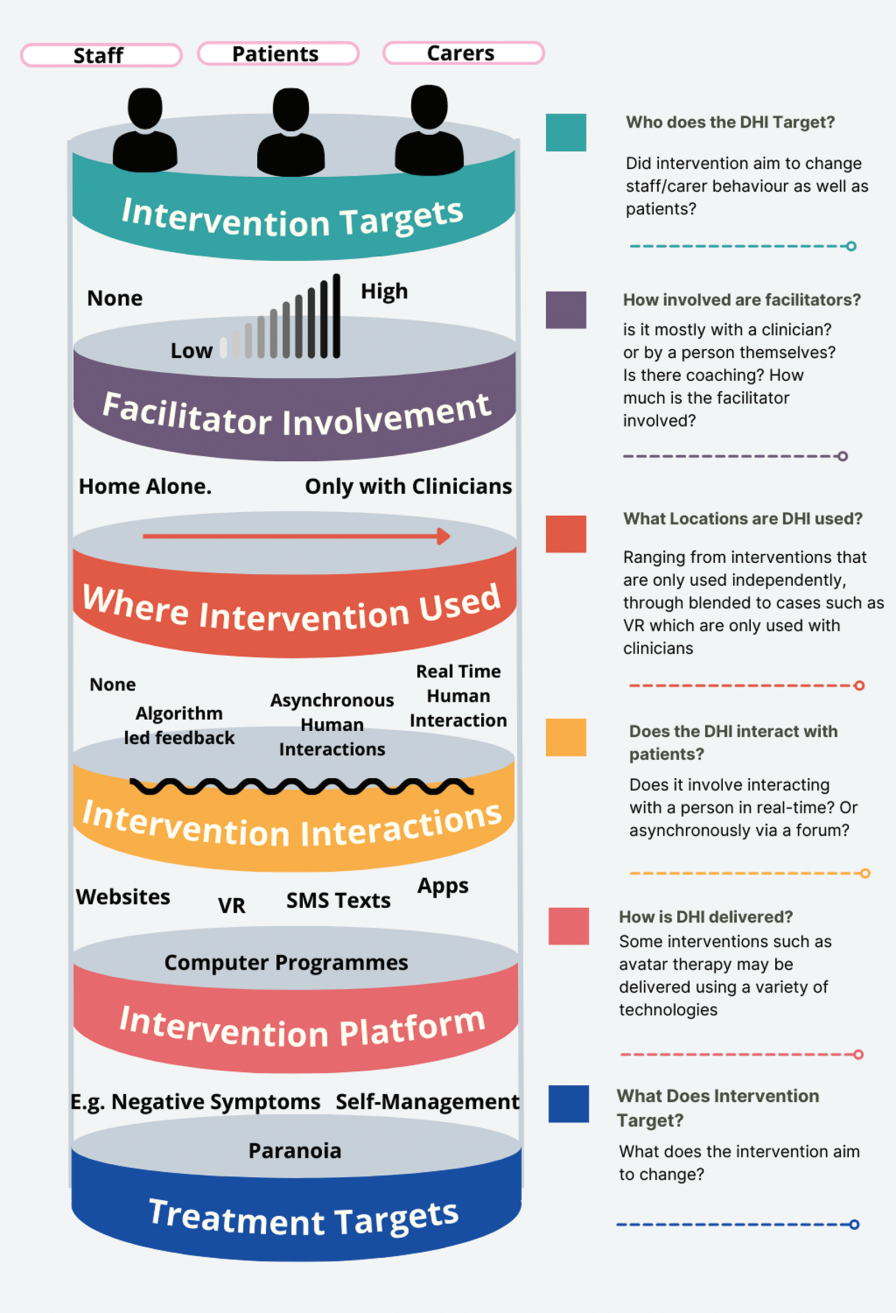
Digital health intervention typology domains.

DHIs described in the individual studies were mapped onto a typology to allow meaningful comparisons between interventions (for the different typology components please see figure 1) and to provide an overview of different intervention types and their key components. Following this, interventions could be classified in a way that is meaningful for comparing AE reports across different forms of DHI; eg, enabling the future comparison of interventions that share an attribute, such as interventions that provided contact with peer support workers compared to interventions that do not. To develop the typology, we examined within and across the descriptions of interventions in the manuscripts of included studies. We considered whether the interventions were different, or if they could be linked by shared attributes. The overarching concepts measured in typological research of health interventions^25^ refer to commonalities shared across different interventions which are meaningful for comparisons. Due to the exploratory nature and lack of preexisting codebook, we presented the initial typology to our diverse research team who critiqued it and suggested missing overarching concepts—this was reiterated over several versions. The interventions were coded for 1 (present) or 0 (absence or not relevant) and are reported in table 2.

**Table 2.** AE Thematic Content From the 1285 Codes (From Original 593 Reported Events)

Adverse Consequence Theme	Description	Overall Occurrence, *n*, (%)	Paraphrased Examples From Dataset	Category
Psychiatric admission	Describes admission either voluntary or involuntary to a psychiatric ward	284 (22.1)	“Patient was admitted to the psychiatric inpatient unit”	Mental healthcare
Unscheduled mental health care (noncrisis)	Requiring increased mental health care, but not at the level of crisis care or psychiatric admission	56 (4.36)	“Participant received closer contact with care coordinator”	Mental healthcare
Crisis care (nonadmission)	Describes accessing crisis care not in an inpatient site	34 (2.65)	“Client accessed crisis team as they were having a rough time”	Mental healthcare
Psychosis exacerbation	Describes an increase in psychotic symptoms	281 (21.9)	“Increasing expression of psychotic symptoms, stating that animals were coming out of parts of the body”	Psychosis symptoms
Affective exacerbation	Describes an increase in affective symptoms	132 (10.3)	“Participant stated they are having a lot of anxiety”	Affective symptoms
Physical health admission	Describes an admission to a hospital for physical health problems	69 (5.37)	“Participant admitted to Cardiology department”	Physical healthcare
Physical health treatment	Describes unscheduled physical health treatment	51 (3.97)	“Attended Accident and Emergency for treatment for physical health problem, discharged”	Physical healthcare
Substance misuse	Describes using nonprescribed psychoactive substances	70 (5.45)	“Participant was using marijuana and meth”	Substance misuse
Suicide ideation	Describes patient experiences of suicidal ideation	99 (7.70)	“Participant had suicidal ideation”	Suicide and non-suicidal self-injury (NSSI)
Suicide plan	Describes suicide planning	26 (2.02)	“Participant reported a plan to end their life”	Suicide and NSSI
Self-harm	Describes self-harm not in the context of a suicide attempt	31 (2.41)	“Participant reported self-harm by cutting”	Suicide and NSSI
Suicide attempts	Describes suicide attempts	13 (1.01)	“Participant attempted suicide by overdose”	Suicide and NSSI
Death by suicide	Describes deaths due to suicide.	1 (0.07)	“Participant died by suicide”	Death
Death (Other)	Describes deaths not due to suicide	2 (0.15)	“Participant died due to medical problems”	Death
Interactions with criminal justice system	Describes interactions with the police	44 (3.42)	“Clinic staff called police who took patient to hospital due to increased aggression”	Legal system
Harm to others	Reports of a patient harming another person	33 (2.57)	“Collateral obtained from patient’s sibling stated that patient had been threatening to harm them”	Harm
Harm from others	Describes a patient being abused by another person	6 (0.47)	“Participant described being attacked unprovoked on the street by a stranger”	Harm
Stopping psychiatric medication	Describes a patient ceasing to take prescribed medication without support from a clinical team	44 (3.42s)	“Abrupt stop to medication regime”	Medication
Other	Idiosyncratic	9 (0.70)	“Participant was yelling while eating”	Other

## Results

### Requests for Individual Level AE Data

**Fig. 2.**
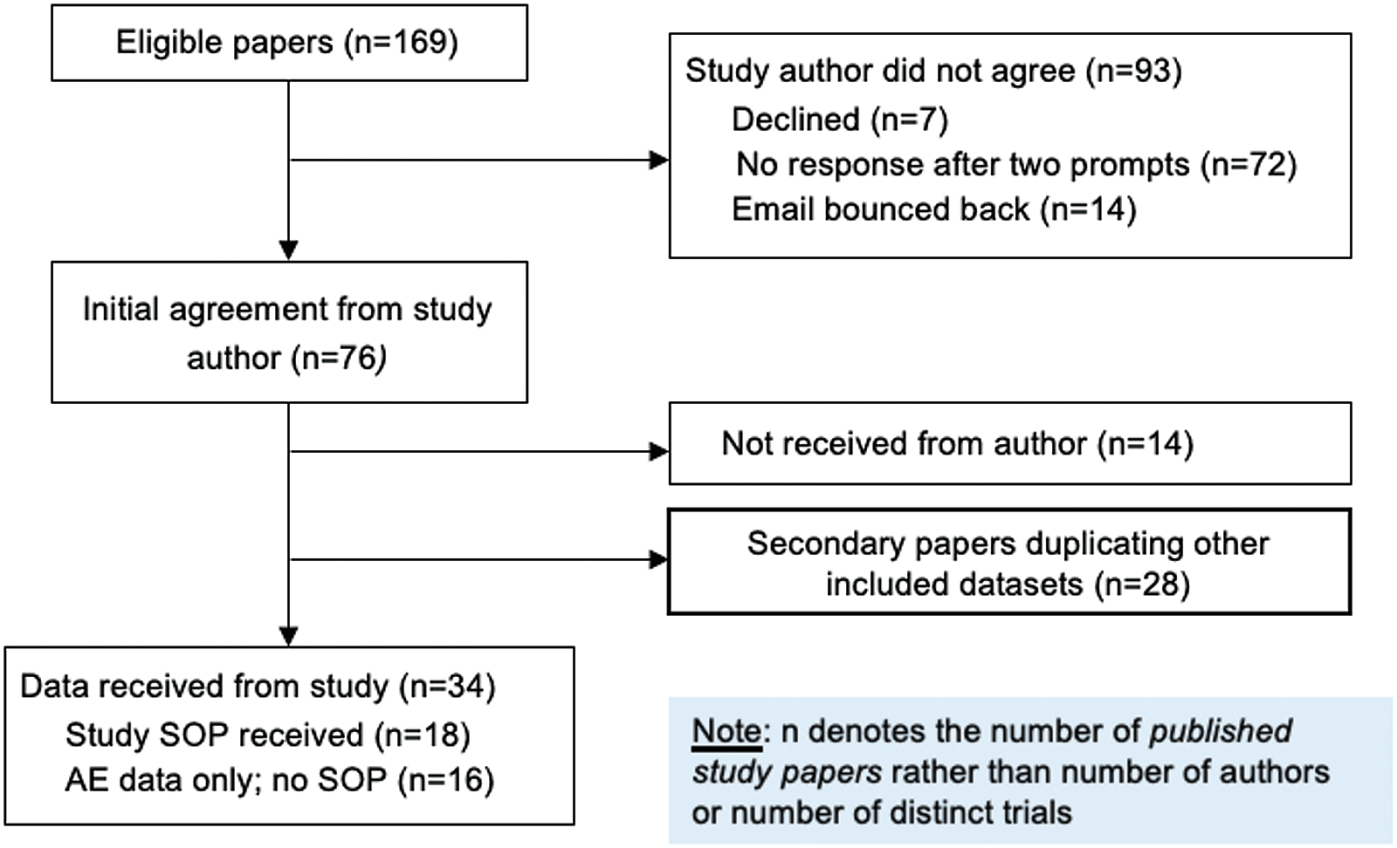
Flow diagram.

Of the 169 authors approached for individual AE level data, 34 shared datasets, of which 18 datasets recorded AEs. No AEs were reported in 16 of the datasets received (see figure 2). 593 raw individual-level AEs were analyzed. Table 1 presents characteristics of included studies for each DHI trial, prevalence of AE thematic codes with the raw percentage for occurrence of different types of AE. As can be seen, the studies represent 1600 people with psychosis. The studies came from 7 countries, most frequently the United Kingdom (*n* = 7 datasets), followed by United States (*n* = 5), Spain (*n* = 2) and 1 study each from Belgium, Australia, and The Netherlands. Smartphones apps were the most common digital platform used (*n* = 13 studies), of which 10 included blended human support ranging from check-in calls to the app being part of therapy. Three studies involved virtual reality-supported therapy. Of note from the application of the typology: 13 studies reported inviting participants to engage in self-monitoring, 10 studies gave software-based feedback, 3 studies reported asynchronous human contact (which could include contact with other patients on forums), and 11 reported synchronous human contact which could include therapy interactions or phone calls from staff. The number of AEs reported across studies ranged from 1 to 274 AEs.

**Table 1.** Characteristics of Included Studies and the Percentage of AE Thematic Codes Identified From the Content Analysis

Author, Year, Country	Name of DHI	Type of DHI	DHI Content	Sample Size	No. AEs Reported and Details If Serious	Sample Recruited	Relatedness	Typology	Percentage of Thematic Codes Identified During Content Analysis
Alvarez-Jimenez et al., 2021, Australia^26^	HORYZONS	Online forum	Website	170	45 SAEs	FEP	No related AEs reported (*n* = 45)	Intended targets: Patients where used: Used at home Facilitator involvement: Asynchronous human feedback Intervention interactions: Peer Contac, Treatment target: Relapse prevention, social functioning, vocational recovery Platform: Website	Psychosis exacerbation: 25.6% Psychiatric admission: 34.1% Suicide ideation: 7.3% Affective exacerbation: 9.8% Physical health admission: 22%
Berry et al., 2021, UK^27^	Assessing measures of physical activity and sleep in schizophrenia	Sleep and physical activity monitoring and feedback via wearables	Wearables	30	12, all AEs	Schizophrenia spectrum disorders	Relatedness not reported (*n* = 12)	Intended targets: Patients Facilitator involvement: None Where used: Used at home Treatment targets: None (exploring acceptability of self-monitoring) Platform: Mobile Phone app, wearable	Affective exacerbation: 50% Psychosis exacerbation: 50%
Bonet et al., 2020, Spain^28^	Remindcare app	Smartphone app	Self-monitoring; Medication reminders	59	25, Of which 12 were AEs and 13 were serious AEs	FEP	Related to intervention (*n* = 2—AEs), related to trial procedures (*n* = 1—SAE), unrelated (*n* = 13) Relatedness not reported (*n* = 9—all AEs)	Intended targets: Patients Facilitator involvement: Asynchronous human contact (mental health staff responded to data indicating clinical change, patients requesting support or discontinuation alerts for patients not using the app) Where used: Home and with Clinicians Intervention interactions: Self-monitoring Treatment targets: Self-management, symptom monitoring Platform: Mobile app	Harm to others: 2.1% Suicide ideation: 2.1% Suicide plan: 2.1% Self-harm: 2.1% Stopping medication: 4.2% Psychiatric admission: 6.2% Crisis care nonadmission: 12.5% Psychosis exacerbation: 39.6% Increased standard mental health care: 29.2%
Bucci et al (*in preparation*), UK	Actissist app; ClinTouch app	Smartphone apps	Actissist: CBT strategies targeting early psychosis relapse indicators. ClinTouch: psychotic and mood symptoms monitoring	172	95, of which *n* = 67 AEs, *n* = 19 serious adverse event, *n* = 10 adverse reaction	FEP (within 5 years of first onset)	0 serious events were related to the study hardware, software, or study assessments. 9 nonserious AEs: Related to software (*n* = 3), related to hardware (*n* = 2), related to study assessments (*n* = 4)	Intended targets: Patients Facilitator involvement: None Where used: Home Intervention interactions: Self-monitoring; Software-based feedback Treatment targets: Self-management Platform: Mobile app	Harm to others: 1.2% Encounters with legal system: 3.1% Other: 3.1% Psychosis exacerbation: 8.6% Psychiatric admission: 6.8% Affective exacerbation: 12.3% Self-harm—9.3% Substance Misuse—6.8% crisis care nonadmission—5.6% Physical health admission: 4.3% Physical health treatment: 14.2% Suicide ideation: 11.1% Suicide plan: 3.7% Increased standard mental health care: 9.3%
Bucci et al., 2018, UK^29^	Actissist app; ClinTouch app	Smartphone apps	Actissist: CBT strategies targeting early psychosis relapse indicators. ClinTouch: psychotic and mood symptoms monitoring	36	6, of which 4 SAEs and 2 AEs	FEP (within 5 years of first onset)	No related AEs reported (*n* = 6)	Intended targets: Patients Facilitator involvement: None Where used: Home Intervention interactions: Self-monitoring; Software-based feedback Treatment targets: Self-management Platform: Mobile app	Crisis care nonadmission: 20% Increased standard mental health care: 10% Harm to others: 10% Psychiatric admission: 10% Psychosis exacerbation: 20% Self-harm: 20% Stopping medication: 10%
Cella et al., 2022, UK^30^	V-NeST: a pilot randomized control trial	VR supported therapy	VR supported therapy (12 sessions)	30	13, of which 11 AEs and 2 SAEs	Schizophrenia spectrum disorders	No related AEs reported	Intended targets: Patients Where used: Used with clinician Facilitator involvement: Synchronous human contact (therapist) Intervention interactions: Therapy Treatment targets: Negative symptoms Platform: VR	Affective exacerbation: 50% Stopping medication: 25% Suicide ideation: 25%
Depp et al., 2019, USA^31^	CBT2Go	Smartphone app	EMA; CBT strategies	255	33—unclear from reporting whether considered AE or SAE	Schizophrenia; Bipolar disorder	Relatedness not reported (*n* = 33).	Intended targets: Patients Facilitator involvement: Synchronous human contact (one-off therapy session, weekly phone calls Where used: Home and with clinician Treatment targets: Self-Management, Defeatist beliefs Platform: Mobile phone app	Psychosis admission: 38.5% Psychosis exacerbation: 38.5% Physical health treatment: 9.6% Physical health admission: 13.5%
Eisner et al., 2019, UK^32^	ExPRESS	Smartphone app	Symptoms monitoring	18	3, all AEs	Schizophrenia spectrum disorders	No related AEs reported (*n* = 3)	Intended targets: Patients Facilitator involvement: Synchronous human contact (phone calls) Where used: Home Intervention interactions: Self-monitoring; Software-based feedback Treatment targets: Symptom monitoring Platform: Mobile phone app	Psychosis exacerbation: 100%
Garety et al., 2021, UK^22^	SlowMo	Smartphone app blended with 8 sessions of CBT	Self-monitoring; Active psychoeducation	361	51, of *n* = 1 adverse reaction, AE *n* = 1, *n* = 1 serious adverse reaction, *n* = 48 SAEs	Schizophrenia spectrum disorders	Related to hardware (*n* = 1—adverse reaction), related to trial procedures (*n* = 1), not related (*n*-47), relatedness uncertain (*n* = 1)	Intended targets: Patients Where used: Used with clinician and at home Facilitator involvement: Synchronous human contact (therapist) Intervention interactions: Self-monitoring; Software-based feedback; Therapy Treatment targets: Paranoia Platform: Mobile phone app	Affective exacerbation: 6.1% crisis care (nonadmission): 5.3% Encounters with legal systems: 7.6% Harm to others: 3.8% Harm from others: 3.8% Increased standard mental health care: 10.6% Other: 1.5% Physical health treatment: 3.8% Physical health admission: 5.3% Psychosis exacerbation: 12.1% Psychiatric admission: 14.4% Stopping medication: 5.3% Self-harm: 2.3% Substance misuse: 9.8% Suicide ideation: 6.1% Suicide plan: 0.8% Increased standard mental health care: 10.6%
Granholm et al, 2020, USA^33^	MA-CBSST	PDA EMI programme	Homework reminders; Behavioral activation	57	3, of which 2 SAEs and 1 AE	Older adults schizophrenia/schizoaffective disorder	No related AEs reported (*n* = 3)	Intended targets: Patients Facilitator involvement: Synchronous human contact (therapist) Where used: Home Intervention interactions: Peer contact (group therapy), Group therapy, prompting Treatment targets: Social skills Platform: PDA	Psychosis exacerbation: 25% Psychiatric hospital admission: 12% Physical health admission: 12% Stopping medication: 25% Suicide ideation: 25%
Granholm et al., 2020, USA^34^	mCBTn	App blended with 12 sessions of group therapy	Mobile App	31	1 SAE	Schizophrenia spectrum disorders	No related AEs reported (*n* = 1)	Intended targets: Patients Where used: Used with clinician and used at home Facilitator involvement: Synchronous human contact (therapist) Intervention interactions: Peer contact; Self-monitoring; Synchronous human feedback (therapy) Treatment targets: Negative symptoms Platform: Mobile phone app	Physical health admission: 33% Psychosis exacerbation: 33% Suicide ideation: 33%
Gumley et al., 2022, UK and Australia^35^	EMPOWER	Smartphone app blended with peer support phone calls	Active symptom monitoring	73	11, of which 10 AEs and 1 SAE	Schizophrenia spectrum disorders	Related to intervention software (*n* = 10), related to hardware (*n* = 1)	Intended targets: Patients, carers, and mental health staff Facilitator involvement: Synchronous human contact (peer support worker phone calls) Where used: Home and during routine mental health encounters. Intervention interactions: Self-monitoring; Peer support contact; Software-based feedback Treatment targets: Self-management, relapse prevention. Platform: Mobile phone app	Affective exacerbation: 64.3% Physical health admission: 7.1% Psychosis exacerbation: 14.3 %
Homan et al., 2020, USA^36^	ICRC program; 4 interventions	Individually tailored combination of: smartphone app, daily support website, online CBT modules, prescriber decision support system algorithm	Self-monitoring on an app; Website; Computer program	462	274 SAEs	Schizophrenia spectrum conditions	Relatedness not reported (*n* = 274, all serious AEs).	Intended targets: Patients and mental health staff Where used: Used at home and with clinicians Facilitator involvement: Synchronous human contact (therapist), asynchronous peer contact (forum) Intervention interactions: Therapy, software-based feedback, self-monitoring Treatment targets: Relapse prevention Platform: Website, Mobile app, Computer program (delivered via website) Staff: Computer program	Physical health admission: 3.7% Physical health treatment: 2.3% Substance misuse: 6% Suicide ideation: 8.2% Suicide plan: 2.3% Self-harm: 1.4% Suicide attempt: 1.5% Affective exacerbation: 10.5% Psychosis Exacerbation: 23% Increased standard mental health care: 1.6% Crisis care (nonadmission): 1.3% Psychiatric admission: 26.3% Encounters with legal systems: 3.9% Harm to others: 3.2% Harm from others: 0.1% Other: 0.2%
Myin-Germeys et al., 2022, Belgium^37^	INTERACT	Smartphone app blended with therapy	Acceptance and commitment therapy strategies	148	5 SAEs	Early psychosis	Related to trial procedure (*n* = 1), unrelated (*n* = 3) Relatedness not reported for *n* = 1 AE All were serious AEs (*n* = 5)	Intended targets: Patients Where used: Used with clinician and at home Facilitator involvement: Synchronous human contact (therapist) intervention interactions: Self-monitoring; Software-based feedback, therapy Treatment targets: Reducing distress Platform: Mobile phone app	Affective Exacerbation: 9.1% Death by suicide: 9.1% Psychosis exacerbation: 18.2% Physical health treatment: 9.1% Psychiatric admission: 27.3% Stopping medication: 9.1% Suicide ideation: 9.1% Suicide panning: 9.1%
Pot-Kolder et al., 2018, Netherlands^38^	VR-based cognitive behavioral therapy for patients with psychotic disorders	VR supported therapy	VR supported therapy (16 sessions)	116	2 SAEs	Psychotic disorders	No related AEs reported (*n* = 2, serious AEs)	Intended targets: Patients Where used: With clinician Facilitator involvement: Synchronous human contact (therapist) Intervention interactions: Therapy Treatment targets: Paranoia (positive symptoms) and safety behaviors Platform: VR	Death (not suicide): 100%
Rus-Calafell et al, 2014, Spain^39^	Social skills training	VR supported therapy	VR supported therapy (16 sessions)	12	11 AEs	Schizophrenia spectrum disorders	No related AEs reported (*n* = 12)	Intended targets: Patients Where used: With clinician VR; Synchronous human; Feedback (therapy) Treatment target: Social Skills Platform: VR	Psychosis exacerbation: 100%
Thonon et al, 2022, USA^40^	A group intervention for motivational deficits: preliminary investigation of a blended care approach using ambulatory assessment	Smartphone app blended + group therapy	ESM supported therapy	8	2 AEs	Schizophrenia spectrum disorders	No related AEs reported (*n* = 2)	Intended targets: Patients Where used: Used with clinician and at home Intervention interactions: Self-monitoring; Peer contact (during group therapy); Therapy Treatment targets: Social skills Platform: Mobile app, wearable	Psychiatric admission: 50% Substance misuse: 50%
von Malachowsk et al., 2022, Germany^41^	IMPACHS	Smartphone app	CBT courses; Self-assessment	24	1 AE	Schizophrenia spectrum disorders	No related AEs reported (*n* = 1)	Intended targets: Patients and staff Facilitator involvement: Synchronous human contact (therapist) Where used: Home and with clinicians Intervention interactions: Software-based feedback, Self-monitoring; Therapy treatment targets: Self-management, general psychosis symptoms Platform: Mobile app Staff platform: Website	Psychiatric admission: 100%

*Note*: ARs, adverse reactions; CBT, cognitive behavior therapy; EMA, ecological momentary assessment; EMI, ecological momentary intervention; ESM, experience sampling methodology; FEP, first episode psychosis; ICRC, improving care and reducing cost; MA-CBSST, mobile-assisted CBSST intervention; mCBT, mobile-assisted cognitive behavioral therapy for negative symptom; PDA, personal digital assistant; UK, United Kingdom; USA, United States of America; V-NeST, virtual reality supported therapy for the negative symptoms of psychosis; VR, virtual reality.

### Overview of Results in Relation to Thematic Codes

The AE coding framework developed in this study comprised 19 thematic codes shown, with definitions and examples in table 2 and summarized here: *n* = 284 psychiatric admission (22.1%), *n* = 281 psychosis exacerbation (21.9%), *n* = 132 affective exacerbation (10.3%), *n* = 99 suicide ideation (7.70%), *n* = 70 substance misuse (5.45%), *n* = 69 physical health admission (5.37%), *n* = 56 unscheduled mental health care—noncrisis (4.36%), *n* = 51 physical health treatment (3.97%), *n* = 44 interactions with criminal justice system (3.42%), *n* = 44 stopping psychiatric medication (3.42%), *n* = 44 crisis care (nonadmission, 2.65%), *n* = 33 harm caused by the participant to other people (2.57%), *n* = 31 self-harm (2.41%), *n* = 26 suicide plan (2.02%), *n* = 13 suicide attempts (1.01%), *n* = 9 other (0.70%), *n* = 6 harm to participants from others (0.47%), *n* = 2 death (other—0.15%), and *n* = 1 death by suicide (0.07%).

As individual AEs (*n* = 593) could be associated with multiple codes to reflect additional context in the provided text description, a total of 1285 codes were developed. The overall occurrence shows how many times the AE occurred in the dataset, and the percentage of AEs in total reported in the dataset. The type of AEs recorded in retrieved datasets included hospital admissions (both psychiatric and general hospital), unscheduled and crisis mental healthcare, symptom exacerbation (including psychosis and physical health related), substance misuse, suicidal ideation/behavior, death, interactions with the criminal justice system, harm (to/from others), and AEs related to psychiatric medication. As anticipated, psychiatric admission and psychosis exacerbation were the most frequently coded AE, comprising just under 45% of the total. However, the fact that individual AEs could be associated with multiple codes allowed consideration of contextual factors such as affective exacerbation, suicidal ideation, and substance use which might be overshadowed in standard reporting.

### Relatedness

Of the 18 DHIs studies examined, 5 (27%) did not provide data on relatedness for all (*n* = 2) or at least some AEs (*n* = 2), with another being unclear (*n* = 1). Ten DHIs (55%) reported no AEs that were rated as “related to trial procedures,” with a further 5 reporting some related AEs in addition to unrelated AEs. Five (27%) reported AEs that were related to the study in some way. At the level of frequency of AEs, of the original 593 events, 29 (4.9%) were reported by the authors as being related to trial participation. From this, we considered the following 4 types of relatedness within the dataset, noting that some AEs featured evidence of relatedness across more than 1 category (eg, reporting a negative reaction to trial procedures in addition to intervention software, which resulted in an increased number of relatedness reports):

Related to the trial procedure. This described cases where the AE was associated with events such as taking part in a trial baseline assessment. In total, 9 (1.5%) of the AE reports featured this. From the thematic codes, these ranged from affective exacerbations (*n* = 4), psychosis exacerbation (*n* = 2), substance misuse (*n* = 2), physical healthcare (*n* = 1), and physical health hospitalization (*n* = 1) and a single psychiatric hospitalization (*n* = 1). In this case, the psychiatric hospitalization was described as due to the assessment bringing up difficult experiences. Of note, there were 2 cases where a participant required physical health care. One case was related to a negative reaction to a functional magnetic resonance imaging (fMRI) scan and another from a physical reaction to feeling overwhelmed.
Related to therapy. This described cases where the AE was related to the therapy element of an intervention which included blended human support. No codes were identified that were related to this (0%).
Related to DHIs. This described cases where the AE was related to software features such as when patient interaction with a DHI resulted in an intervention action. In total, there were 16 AE reports (2.7%) associated with the DHI itself. When looking at the thematic codes from the analysis, these appeared to be associated with affective exacerbation (*n* = 10), some psychosis exacerbation (*n* = 7), and stopping using medication (*n* = 2). In another case, a participant required physical health admission due to feeling overwhelmed by the DHI.
Related to hardware. This described cases where the AE was related to the hardware that delivered the DHI *such as participant experiences of using mobile phones.* There were 6 hardware-related AE reports (1.0%). From the thematic codes, these appeared associated with affective exacerbation (*n* = 3), psychosis exacerbation (*n* = 2), and other (*n* = 2).

### Overview of Results in Relation to Intervention Typology

The related 16 AE reports that were related to the DHI came from 3 DHIs that utilized self-monitoring on mobile phone-based apps. The interventions were blended with human support (*n* = 2) or stand-alone (*n* = 1).

## Discussion

In this study, an international network conducted an individual-level content analysis to quantify and characterize the types of AEs occurring in DHI trials for psychosis. This is, to date, the most in-depth exploration of AEs in DHIs for psychosis. The resulting framework comprised 19 codes, including less overt negative consequences and contextual factors which are impactful for patients but may be overshadowed within existing monitoring and reporting procedures (eg, affective exacerbation). Five studies only reported data that could be considered a serious AE such as hospitalization or death—this has been noted in other psychological intervention research^13^ and carries the risk that related negative consequences which are potentially important to the person experiencing them, may be missed. The analysis showed that only 4.9% of reports from 18 DHIs were related, providing evidence in support of the safety of the interventions for which data was provided. Of the 2.7% related to the DHI, not including hardware such as finding beeps from the phone distressing, the related negative DHI consequences were affective exacerbation, psychosis exacerbation, people stopping using prescribed medication, and in 1 instance someone needing a physical health admission due to feeling overwhelmed. During the systematic search, a further 16 studies reported no AEs. The results suggest that affective and psychosis exacerbations linked to usage of DHIs may be expected, at least in a minority of patients. For example, reports from a trial of a self-monitoring intervention designed to increase patient awareness of their mental state by generating charts showing symptoms over time that could be discussed with clinicians and peer support workers^35^ identified some mild increases in distress (both mood changes and increased some psychosis) linked to this intervention mechanism. However, the identification of these milder exacerbations may be due to the rigor of the AE monitoring employed during the trial. Taken together, these results suggest that negative consequences may be rare in DHI evaluations. However, over one quarter of studies did not consistently report whether the AE was related to the DHI. While this data may have been collected in the trial but not shared for the purpose of this study, it suggests the importance of ensuring that the issue of relatedness is prioritized within standardized operating procedures for AE monitoring.

To assist researchers in determining whether specific AEs may be anticipated given a particular intervention, a dark logic model may be employed.^18^ This involves researchers directly assessing where the interventions’ theory of change may result in AEs.^42^ For example, a dark logic model could identify the mechanisms by which the intervention target of self-monitoring may result in a temporary increase in worry. The coding framework developed in this study facilitates differentiation of AEs which are “mechanism related” (linked to underlying program theory) from more general worries associated with accessing a DHI such as worries about struggling to use a digital device. Consulting AE reports from preexisting interventions that use similar components, through typologies such as that proposed in table 1, may be a useful means of identifying patterns in AEs linked to specific digital functionality and intervention targets.

In addition to the missing data on relatedness, several AEs analyzed from the available datasets were unclear; eg, reports which refer to patients being “hospitalized” but with no specification in terms of physical or mental health admission or relatedness. This suggests the need for improvement and standardization in the reporting of AEs in the context of DHIs, which in turn requires enhanced rigor in standard operating procedures (SOPs) relating to the monitoring of AEs within trials. As has been indicated in initiatives in autism research,^43^ strategies to improve the reporting of AEs need to address limitations including a failure to consider AEs in initial study design, inadequate methods of collecting data, and lack of transparency in the reporting of data. Only 20% of authors who were asked to share AE data did so, which suggests restricted data reporting may be common. Cooperation among researchers by adhering to open science principles through sharing anonymized AE data (as has been the case in this study) would make conducting individual-level analyses of intervention safety easier to achieve by addressing issues such as academic precarity which can act as a block to data sharing when researchers stop working within time-limited projects. As has been noted elsewhere, this would also reduce the ability of authors to potentially “cherry pick” the reporting of AEs.^44^ The harms extension of the Consolidated Standards of Reporting Trials statement (CONSORT)^45^ provides 10 recommendations on best practice reporting of AEs and can be used to guide AE reporting in clinical trials. Furthermore, our iCharts consortium have made specific recommendations and published guidance on monitoring, recording, and reporting AEs in the context of DHI trials,^23^ including suggested methods to consider for eliciting AE reports, recommendations on researcher training and supervision, eg, AE reporting forms (detailing a description of the AE, causality, relatedness, severity, seriousness), and a template SOP.

While more robust monitoring may give a more comprehensive view of AEs, clear identification of staff responsibilities to enquire about and elicit AE reports. Purposively sampling dropouts within qualitative studies exploring the experience of engaging with DHIs may elucidate links between AEs and intervention disengagement and allow researchers to better comment on overall acceptability and feasibility. Enabling participants to report how an app is impacting their mental health through direct questioning by specially trained and unblinded researchers, or regular check-in conversations with research staff (including peer support workers) such as in the case of the EMPOWER intervention,^35^ may enable detection of events which could be missed through standard AE monitoring. Focusing on the patient perspective may address the epistemic injustice faced by people with psychosis.^46^ Epistemic injustice describes discrimination against marginalized people as “knowers” and comprises 2 facets that are relevant to the consideration of AEs: testimonial injustice, where patients’ reporting of an intervention causing distress is dismissed, and hermeneutical injustice—defined as when patients are not able to defining what an AE means for them.^47^ The issue of underreporting of nonserious AEs has been noted in pharmaceutical clinical trials.^48^ Self-reporting of AEs has been encouraged in oncology trials^49^ and through the “yellow card” scheme in the United Kingdom.^50^ Providing a way for patients to self-report AEs may increase the quality of information available to researchers and bring greater understanding around potential harms of the intervention under investigation and could be implemented within interventions such as apps—many of which already encourage self-monitoring. However, any methods would need to be systematically tested to understand potential sources of bias. The codes identified in the content analysis may be helpful in developing automated systems to detect AEs in direct patient reports^51^ because they identify issues that are likely to impact patients, such as increased anxiety. Such issues may be missed by relying on medical reports which come about when distress reaches a level requiring professional input. Access to such data would help determine the longevity of any adverse responses and help clinicians support patients to weigh up the benefits and potential negative impacts when considering using a DHI.

In moving the field toward standardized AE monitoring, we would expect that DHI trials employing more robust monitoring would generate higher frequencies (as a function of this robustness) when compared with earlier studies. This is an important point to recognize within the wider community to avoid negative comparisons between future trials and earlier trials employing less comprehensive methods. Access to comprehensive AE data would enable research focused on clustering techniques to identify similarities that can be seen in other medical fields.^52^ Any analysis of data from novel monitoring tools will also need to delineate the specific risks associated with DHIs from general risks associated with noninterventional technology usage, given the active research into the potential impact of screen time and smartphone use on the developing brain and psychosis.^53,54^ We make specific recommendations in table 3.

**Table 3.** Adverse Event Reporting Recommendations

Recommendations	Rationale
*Recommendation 1.* Studies should report the data generating process underpinning AE collection as part of the AE report in terms of how: (1) AEs were gathered, and details of training provided to staff in gathering AEs; and (2) AE data is accessed such as by reporting frequency of patient contacts or how often clinical notes are accessed. The iCharts consortium have produced best practice guidelines.^23^	From assessing AE reports, it can be unclear how the AE reports were gathered as this information was not routinely reported. For example, it was not always clear whether researchers asked participants about AEs directly, or whether AEs were only logged when spontaneously reported or gathered from case notes.
*Recommendation 2.* Studies should plan from the start that AE reports form part of the outcome data presented in trial reports and highlight to participants that AE data might be shared with other researchers. For example, consent forms should highlight participants to the fact anonymized safety information will be shared with other researchers.	There was a low response rate to requests made by the iCharts consortium to researchers asking them to share AE reports. There may be many reasons for this. Data sharing initiatives usually focus on primary outcome measures and AE reports may not have been traditionally considered as data that can be shared with other researchers.
*Recommendation 3.* Researchers should ensure that all AE reports are written in enough detail so that independent researchers can understand what happened to the participant when fully anonymized reports are shared and how many participants were affected. For example, if a participant was hospitalized—was this for physical or mental health care? If this was for mental healthcare, was this informal or under section?	Around 1 in 10 AE reports were unclear which meant it could not be clearly determined what had happened to participants. Improvement would be best achieved through training the whole study team in a standardized approach to adverse event monitoring. We discuss this in more detail elsewhere.^23^
*Recommendation 4.* Expanding upon Recommendation 3, Researchers should report whether the AE was related to study participation and provide sufficient detail for an independent observer to understand the context of the AE and the evidence for rating of relatedness.	From appraising the AE reports, it was clear that AEs could be related to many different parts of study participation. Reporting this in further detail will help determine research safety.

### Strengths and Limitations

We analyzed individual-level patient data and developed a coding framework that was co-produced with patients and mental health staff to code extant AEs. The resulting framework included contextual factors that are often overshadowed by hospital admission within standard reporting. However, there are limitations. First, AEs included in the analysis are only a comparatively small subset of published DHI psychosis trials, potentially limiting, and biasing our findings. Second, the early stage of the field means there was high heterogeneity in how AEs were defined, monitored, and recorded. Third, individual level AE data was not split according to treatment arm (ie, DHI treatment arm vs Treatment and Usual/ control arm). Therefore, we cannot be certain whether the AEs recorded in our analysis related to the DHI itself or AEs recorded in the control condition. Future studies should explore AEs across different intervention arms employing more uniform reporting standards to allow more accurate estimates of expected frequencies of AEs linked to DHIs, with the control arm providing a comparison group. Fourth, UK datasets were over-represented. Fifth, we were reliant on study authors sending us anonymized AE trial data, potentially introducing selection bias by omitting other trials related to AE reporting in other contexts/countries. Lastly, we have included heterogeneous intervention types as we are providing an overview of the current field. Future research should explore qualitative differences between different DHI types such as apps or VR.

## Conclusions

Overall, the findings provide evidence in support of the safety of DHIs for which data was provided. However, significant variability in data quality was observed with around 1 in 10 AE reports classified as being unclear from the raw data and a quarter of studies not providing clear data on relatedness. This highlights a need for standardized monitoring and reporting of AEs to deliver robust evidence on the potential specific harms related to DHIs in psychosis. We have developed specific guidance and templates for enhancing measuring and reporting AEs in DHI trials (see Eisner et al.^23^). It is important for all stakeholders to recognize that standardized rigorous monitoring and reporting will inevitably increase the number of AEs identified within trials. As such, we advocate for a fundamental shift in AE reporting culture where AEs are viewed not as a negative outcome of a research trial, but as a valuable outcome measurement that can enable both clinicians and patients to make informed decisions about DHIs.

## Supplementary Material

Supplementary material is available at https://academic.oup.com/schizophreniabulletin/.

sbae031_suppl_Supplementary_Material
