# Supplementary material for: Adverse Events Reporting in Digital Interventions Evaluations for Psychosis: A Systematic Literature Search and Individual Level Content Analysis of Adverse Event Reports
Source: Schizophr Bull. 2024 Apr 6;50(6):1436–55. doi: 10.1093/schbul/sbae031 (PMC11548921; doi:10.1093/schbul/sbae031)
Supplement: sbae031_suppl_Supplementary_Material [file sbae031_suppl_supplementary_material.docx]

**Supplementary Methods**

***Supplementary methods SM1: Additional details of systematic literature search***

*Overview of search strategy*

For pragmatic reasons, we combined systematic searches from two previous projects led by members of our network. All searches were restricted to peer-reviewed articles published in English between January 2010 and the present day with human participants. The symbols ‘?’ and ‘*’ are wildcards representing one (‘?’) or more (‘*’) characters, respectively.

*Search 1: Barriers and Facilitators review search (search date: 11/10/21)*

Seven databases were searched (MEDLINE, PsycINFO, PsycARTICLES, Embase, Health and Psychosocial Instruments, PubMed, Web of Science). As this search was from a systematic review relating to barriers and facilitators of engagement with digital health tools across SMI diagnoses, it included additional search terms (e.g. bipolar). Overall search terms were as follows:

(schizophren* or schizo?affective or psychotic or psychos?s or paranoia or paranoid or hallucination* or delusion* or bipolar or SMI or serious mental illness or severe mental illness or manic or mania or grandios* or serious mental health or severe mental health or EIS or Early intervention service? or Early intervention team? or early intervention program? or CMHT? or community mental health team? or assertive outreach team? or AOT? or home treatment team? or home based treatment team? or crisis team?).ti,kw,ab.

AND

(digital or m?health or e?health or phone? or smart?phone? or mobile? or app? or application? or wearable? or online or internet or cyber or web or mtherap* or etherap* or Technology or technologies or technological or webpage or website or smart?watch or smart?watches or AI or artificial intelligence or machine learning or smart?device? or digital phenotyping or digital phenotype? or sensing or passive data or passive monitoring or GPS or global positioning system or gyroscope? or accelerometer?).ti,kw,ab.

AND

(barrier? or facilitator? or enabler? or obstacle or implement* or engage* or adhere* or satisfaction or acceptability or usability or user experience? or satisfaction or usage or adoption or user perspective? or qualitative or interview or focus group or feasibility or Co-design).ti,kw,ab.

*Search 2: Virtual Reality study search (search date: 29/04/22)*

Seven databases were searched (MEDLINE, PsycINFO, PsycARTICLES, Embase, Health and Psychosocial Instruments, PubMed, Web of Science) using the following terms:

(schizophren* or schizo?affective or psychotic or psychos?s or paranoia or paranoid or hallucination* or delusion*).ti,kw,ab.

AND

(virtual reality or VR).ti,kw,ab.

***Supplementary methods SM2: PICO criteria***

*Participants/population*

Included

- People aged 16 or over with psychosis or a schizophrenia spectrum diagnosis (e.g. schizophrenia, schizoaffective, schizophreniform, psychosis not otherwise specified).

*Intervention*

Included

- Studies testing the actual use of digital health tools that aim to monitor or improve the mental or physical health of people with a psychosis or schizophrenia spectrum diagnosis
- Delivered using a device such as a smartphone app, text messaging, online/website, virtual reality (VR), or wearable device.

Excluded

- Digital tools used as a component during in-person sessions with no remote use outside these sessions*
- Digital tools used purely for research purposes (with no likely eventual clinical application)
- Only included video-conferencing or phone calls
- Served only as an appointment booking system for in-person therapy
- Electronic health records that only health professionals could view and contribute to
- Only used to screen for the presence of a mental health condition
- Harvested existing data from electronic health records or mainstream social media to make predictions or classifications with regards to mental health.

*With the exception of VR studies, which were included even if they were used during in-person sessions only. The rationale for this was that VR headsets are not commonly owned by the general public but may be in the future. Studies to date are therefore unlikely to include remote use but this is something that may be done in the future.

Comparison

- No comparison group needed

Outcomes

- No specific outcome needed

***Supplementary methods SM3: Email template requesting initial information***

Dear [name],

I hope you don't mind me emailing you out of the blue.

I am contacting you on behalf of our recently established [Research Harmonisation Group](https://schizophreniaresearchsociety.org/2021-rha-winner/). We were awarded the 2021 Schizophrenia International Research Society (SIRS) research harmonisation award, the theme of this year’s award being *Digital Health*. I am delighted to be convening a fantastic group aiming to produce a freely-available tool, reporting procedure and training resource to allow consistent and systematic reporting of adverse events in this context.

We have collated a list of important digital psychosis studies that have collected and reported adverse events data. We note your publication where you are the first/corresponding author:

- [Article citation]

**As a key member of the digital health for psychosis research community, we warmly invite you to join our consortium,** where we aim to catalogue and collate adverse events reported in a digital psychosis context, with the end goal of developing an adverse events reporting procedure, tool and training guidelines. As a consortium member, these resources would be shared with you to use in your ongoing digital health work.

I attach a more detailed proposal about what would be involved if you agree to join the consortium.

In brief, we are asking you to share two things:

1. A **de-identified list** of the type and nature of adverse events reported in your study (this could just be a simple list/table of de-identified adverse events recorded).
2. The **Standard Operating Procedures (SOPs)/procedure/guide** you used to collect adverse events data in your study.

We have developed a simple Qualtrics Form to capture this information, in the hope it would make it as easy for you as possible to share the list.

Please let me know if you have any questions. If you are willing to share the list of adverse events and procedure reported in your study, we will send a link to the Form for completion.

Many thanks in advance for considering this.

Yours sincerely,

Sandra Bucci

*SIRS Research Harmonisation Group Convener, 2021-2023*

***Follow up email***

Dear [name],

I am contacting you on behalf of Professor Sandra Bucci to follow-up on her email inviting you to contribute adverse events data to the iCharts (International Collaboration for Harmonising Adverse Events Reporting in Technology for Serious Mental Health Problems) consortium.

We have developed a simple Qualtrics form where you can upload this information in any format convenient to you: [web link to Qualtrics form]. Further information (including examples) can be found on the link, and the proposal attached.

Please let me know if you have any questions about the same.

Best wishes,

[name]

*Research Assistant | Division of Psychology and Mental Health | University of Manchester*
